# SDK1-ALK Fusion in a Lung Adenocarcinoma Patient With Excellent Response to ALK Inhibitor Treatment: A Case Report

**DOI:** 10.3389/fonc.2022.860060

**Published:** 2022-03-04

**Authors:** Lin Ma, Junjuan Xiao, Yaping Guan, Dongfang Wu, Tiantian Gu, Jun Wang

**Affiliations:** ^1^ Department of Oncology, The First Affiliated Hospital of Shandong First Medical University & Shandong Provincial Qianfoshan Hospital, Jinan, China; ^2^ Shandong Lung Cancer Institute, Jinan, China; ^3^ Shandong Key Laboratory of Rheumatic Disease and Translational Medicine, Jinan, China; ^4^ YuceBio Technology Co., Ltd, Shenzhen, China

**Keywords:** non–small-cell lung cancer, ALK rearrangement, ALK inhibitor, SAF-189s, SDK1-ALK

## Abstract

**Background:**

Rearrangements of Anaplastic lymphoma kinase (*ALK*) have been discovered as a novel driver mutation in patients with non–small-cell lung cancer (NSCLC). Patients’ responses to ALK tyrosine kinase inhibitors (TKIs) may vary depending on the variations of *ALK* rearrangements they have. It is imperative for clinicians to identify druggable *ALK* fusions in routine practice.

**Case Presentation:**

In this study, we discovered a rare *ALK* rearrangement type (*SDK1–ALK*) in a Chinese lung adenocarcinoma patient who responded well to ALK inhibitor SAF-189s. The positive expression of ALK in lung biopsy tissue was verified by IHC analysis. A new *SDK1-ALK* fusion was discovered using NGS. The patient was treated with SAF-189s (160 mg per day) as a first-line therapy and went into continuous remission, with a 12 months progression-free survival at the last follow-up.

**Conclusion:**

This is the first case of *SDK1-ALK* fusion with an excellent response to an ALK inhibitor, which will provide better understanding of ALK-TKI applications for NSCLC patients with *ALK* fusion in the future.

## Introduction

Anaplastic lymphoma kinase (*ALK*) rearrangements account for approximately 5% of patients with non-small-cell lung cancer (NSCLC) and represent as a critical therapeutic target in clinical practice (1). Patients with *ALK* rearrangements in NSCLC are usually younger and light smokers. ALK tyrosine kinase inhibitors (TKIs) provide a marked objective response rate (ORR) and impressive clinical benefit for patients with *ALK*-rearranged lung cancer. The first-generation ALK inhibitor was crizotinib, developed after the discovery of chromosomal rearrangement involving the ALK and echinoderm microtubule-associated protein like 4 (EML4) genes in NSCLC in 2007. Second-generation inhibitors including ceritinib, alectinib, and brigatinib, were authorized for treatment in ALK-positive patients after then. As third-generation inhibitors, lorlatinib and ensartinib have been developed for the treatment of NSCLC patients who have acquired resistance to prior ALK inhibitor treatment (2). In 2021, the FDA has expanded the approval for lorlatinib to include an new indication for the first-line treatment of patients with ALK-positive NSCLC (3). In our case, SAF-189s is a new ALK inhibitor that has the ability to overcome various resistance mutations.

Numerous *ALK* fusion partner genes, such as *EML4 (94%)*, *KIF5B (1.6%)*, and other variants (4.7%), have been identified with the rapid development and application of next-generation sequencing (NGS) (4). For patients who are using ALK inhibitors, distinct fusion patterns are linked to varying clinical outcomes (5). It is imperative for clinicians to delineate a “response diagram” of patients with unknown *ALK* fusion variants treated with ALK inhibitors. Here, we describe an undocumented ALK rearrangement (*SDK1-ALK* fusion) in a lung adenocarcinoma patient who exhibited a remarkable response to SAF-189s.

## Case Presentation

**Figure 1:**
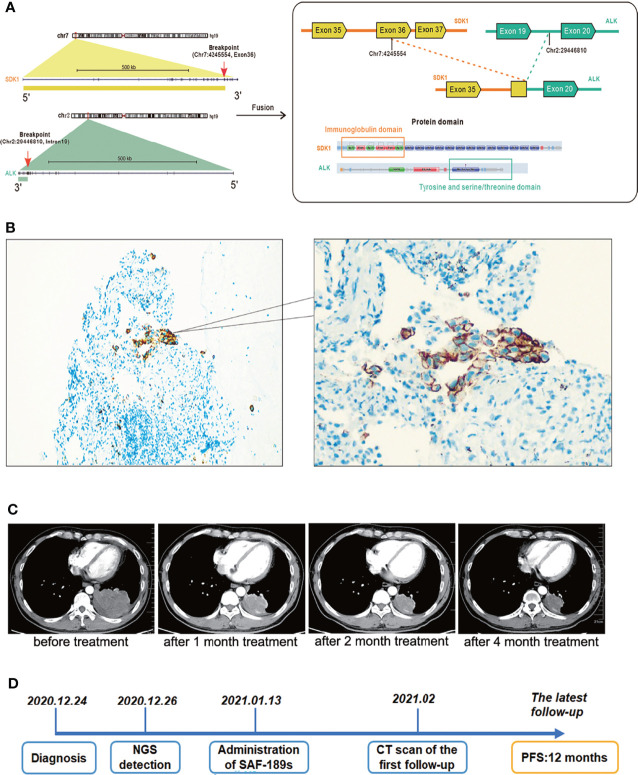
A Chinese patient with adenocarcinoma harboring a novel SDK1-ALK fusion variant exhibited excellent response to ALK inhibition. **(A)** The schematic diagram. **(B)** IHC showed the positive expression of ALK protein. Cancer cells showed an uneven stippled cytoplasmic and perimembranous staining pattern. **(C)** Compared with the baseline images, the size of the lesion in the left lower lobe, as well as lymph nodes in left hilum and mediastinum was significantly reduced after this patient was treated with SAF-189s for 1 month, 2 months, and 4 months. **(D)** The timeline diagram of this case. At the time of the latest follow-up, he tolerated well with only grade 1 rash and had a 12-month progression-free survival. Now he continues to be in partial response and keeps on SAF-189s treatment.

In December 2020, a 47-year-old man with a history of smoking came to our hospital with a paroxysmal dry cough that had lasted about a year and left subcostal pain for two months, which aggravated after deep inhalation. A computed tomography (CT) scan revealed a mass in the left lower lobe measuring 8.1 × 6.1 cm, multiple enlarged lymph nodes in left hilum and mediastinum, obstructive pneumonia in the left lung, and left pleural effusion. Radionuclide bone scan and magnetic resonance imaging revealed no evidence of bone or brain metastasis. His medical history was not remarkable. The pathology of this patient’s lung cancer was verified through a CT-guided percutaneous fine-needle lung biopsy. According to the American Joint Committee on Cancer Staging Manual, 8th edition, he was finally diagnosed with stage IV lung adenocarcinoma (cT4N2M1). The biopsy specimen was subjected to next-generation sequencing (NGS), which revealed a hitherto unknown *SDK1* Exon36-*ALK* Exon20 fusion variant (abundance: 9.2%) (**Figure 1A**). Further immunohistochemical (IHC) analysis indicated the positive expression of ALK protein (clone D5F3, Ventana) (**Figure 1B**).

It is well known that ALK inhibitors, such as Crizotinib and alectinib, are pricey. If the patient had maintained his treatment, he would have been in serious financial trouble. SAF-189s is a new selective ALK inhibitor, and the patient satisfied all of the criteria for clinical trial inclusion. He provided informed permission. On January 13, 2021, the patient was then enrolled onto a phase I/II clinical trial evaluating SAF-189s in NSCLC(Clinical Trials.gov number, NCT00585195) after a thorough enrollment assessment and began with oral administration of SAF-189s at a dose of 160 mg per day. After 10 days of medication, his symptoms considerably improved. On February, 2021, done 2 months after treatment initiation, a chest CT scan revealed a partial response (42 percent reduction in the sum of the target lesion’s longest diameters per RECIST 1.1) (**Figure 1C**), and further radiological assessments confirmed his continuing tumor response to this ALK inhibitor. At the time of the latest follow-up, he tolerated well with only grade 1 rash and had a 12-month progression-free survival (**Figure 1D**). Now he continues to be in partial response and keeps on SAF-189s treatment.

## Discussion

ALK fusions are more common in patient with younger age and light or never smoking history. Like epidermal growth factor receptor (EGFR) mutations that were frequently found in female and adenocarcinoma patients, ALK gene aberrations are routinely found in patients with adenocarcinoma histological subtype. Remarkably, brain metastases are more likely to be found in this subset of patients (6). Many researchers focused on the heterogeneous response mechanism of EGFR-mutated NSCLC patients during EGFR-TKI treatment as the significant proportion of EGFR-positive cases in NSCLC. In patients with *ALK*-rearranged lung cancer, little is known regarding the effectiveness and relevant mechanism of ALK-TKIs. Firstly, different *ALK* fusion partners resulted in different levels of ALK expression and protein stabilities, which may be interpreted as different ALK-TKI sensitivities in individuals (7, 8). Secondly, the signaling networks activated by ALK are intricate due to the various alterations found in individuals. Phosphorylated ERK and STAT3 levels were found to be upregulated in EML4-ALK-positive cell lines. Delineation of the extensive ALK signaling network is critical for the development of ALK inhibitor-based combination therapies.

A number of biomarkers should be tested on account of the incredible accomplishment of precision medicine improving NSCLC patients’ outcomes significantly. Patients with advanced-stage NSCLC always have a limited amount of tissue for molecular analysis. NGS is a valid approach able to detect sufficient gene alterations simultaneously, and it can be started with nucleic acids recovered from patients’ cancer tissues *in situ* or liquid biopsy samples. According to the KWAY Italian multicenter cost evaluation research, the implementation of NGS saves personnel time spent to testing activities and lowers the overall cost of testing per patient (9, 10). Fluorescence *in situ* hybridization and IHC are gold standards for *ALK* mutation testing. NGS further identify the complex *ALK* rearrangement in NSCLC, which can be an effective complement in clinical decision-making and an efficient approach to find novel variants and fusion partners (11).

In this case, NGS was used for the identification of an uncommon *SDK1-ALK* fusion in a patient with lung adenocarcinoma for the first time. SDK, encoded by the *Sidekick* gene was initially discovered in Drosophila, which is one of the largest members in immunoglobulin superfamily (12). *Sdk1 and Sdk2* are vertebrate ortholog of *Sdk*. Genome-wide studies revealed that *SDK1* polymorphism is related to neurological disorders (13, 14). Reports clarified that the *SDK* gene is extremely fragile, and that *SDK* mutations can be found in a variety of human cancers (15, 16). Ren et al. found that *SDK1-AMACR* fusion might be a crucial factor in progression of prostate cancer (17). The specific function of *SDK1* in NSCLC still needs further study. The homophilic binding of Sdk1 ectodomain regions is required for cell-cell aggregation (18). Mutations in *SDK1* gene have been found in the lung tissue of asbestos-exposed patients and cancer specimens of stage I lung adenocarcinoma, which may be responsible for dysfunction of cell adhesion in tumor progression (19, 20). In our case, the *SDK1* Exon36-ALK Exon20 fusion protein contained Sdk1 ectodomain regions and the ALK kinase domain, which might have resulted in ligand-independent dimerization and hence continuous activation of ALK.

Targeted therapy has brought notable clinical benefits to ALK positive NSCLC patients. Crizotinib has become the standard first-line oral TKI therapy in patients with ALK-positive metastatic NSCLC based on the outcomes of the PROFILE 1007 trial and the phase III PROFILE 1014 clinical study. The second-generation ALK TKIs ceritinib and alectinib obtained expedited approval as first-line medications based on the clinical trials ASCEND-4 and ALEX. Another second-generation TKI brigatinib exhibited a 71% ORR in the phase 3 ALTA- 1L trial, making it the new first-line medication for advanced-stage NSCLC patients. Lorlatinib, a third-generation ALK TKI, achieved a 72% improvement in PFS compared with crizotinib as a first-line therapy in the phase III CROWN trial (3, 21). Secondary mutations in the ALK tyrosine kinase domain, such as L1196M, C1156Y, G1202R, and G1269A, are the most common cause of ALK-targeted therapeutic resistance, termed as ALK-dependent resistance. ALK gene amplification and activation of alternative pathways, including epidermal growth factor receptor (EGFR), hepatocyte growth factor receptor, and insulin-like growth factor 1 receptor, represent other resistance mechanisms (2).

SAF-189s is a novel selective ALK inhibitor and can overcome multiple resistance mutation. In a multicenter I/II study, all enrolled 34 ALK-positive patients responded well to SAF-189s treatment, with a confirmed PR of 50% and an unconfirmed PR of 11.7%. In 24 patients who progressed on previous first-line crizotinib or ceritinib treatment, a confirmed PR was 47.6% (22). In the nude mice xenograft model of *SDC4-ROS1* fusion NSCLC, SAF-189s induced tumor regression and exhibited notable prolonged and durable efficacy and was more potent than crizotinib and comparable to lorlatinib against G2032R mutant-driven tumors (23). The present untreated lung cancer patient exhibited excellent response to SAF-189s treatment with manageable toxicities.

Immune checkpoint inhibitors, which can modulate tumoral immunosuppression and reactive host immunity, promising long-term disease control in a segment of patients with advanced NSCLC. As a result, increasing emphasis is being placed on the combination strategies of immunotherapy and targeted therapy. However, the present research findings on ALK TKIs in combination with immunotherapy are still ambiguous. The CheckMate 370 phase 1/2 study and TATTON trial, for example, had halted their enrolments due to severe intolerant toxicities. Conversely, research of alectinib plus atezolizumab showed a manageable side-effects profile with excellent antitumor activity (ORR 85%). The understanding of biological mechanisms underlying immune-targeted combinations still need further clinical investigations and NGS will be a valid tool in future decision-making (24).

## Conclusion

In summary, this is the first case report involving *SDK1-ALK* fusion detected in a Chinese patient with advanced lung adenocarcinoma by using NGS-based cancer genomic DNA profiling in the clinic. In this case, the *SDK1-ALK*-rearranged lung cancer in this patient is susceptible to treatment with a new ALK inhibitor SAF-189s, which is now being studied in a clinical trial. Thus, our study provides a new druggable target for NSCLC driver mutation in routine practice.

## Data Availability Statement

The datasets for this article are not publicly available due to concerns regarding participant/patient anonymity. Requests to access the datasets should be directed to the corresponding author.

## Ethics Statement

The studies involving human participants were reviewed and approved by Ethics Committee of The First Affiliated Hospital of Shandong First Medical University. The patients/participants provided their written informed consent to participate in this study. Written informed consent was obtained from the individual(s) for the publication of any potentially identifiable images or data included in this article.

## Author Contributions

LM: Data curation, Writing- Original draft preparation. JX: Writing - review & editing. TG: Visualization, Investigation. DW: Software. JW: Supervision. All authors contributed to the article and approved the submitted version.

## Funding

This study was supported by the National Natural Science Foundation of China (Grant No. 81572875), CSCO-MSD Cancer Research Foundation (Grant No. Y-MSD2020-0350), CSCO-PILOT Cancer Research Foundation (Grant No. Y-2019AZMS-0440), Wu Jieping Medical Foundation for Clinical Scientific Research (Grant No. 320.6750.2020-12-16), and the Natural Science Foundation of Shandong Province (Grant No. ZR202102190539).

## Conflict of Interest

DW and TG are employees of YuceBio Technology Co., Ltd Shenzhen.

The authors declare that the research was conducted in the absence of any commercial or financial relationships that could be construed as a potential conflict of interest.

## Publisher’s Note

All claims expressed in this article are solely those of the authors and do not necessarily represent those of their affiliated organizations, or those of the publisher, the editors and the reviewers. Any product that may be evaluated in this article, or claim that may be made by its manufacturer, is not guaranteed or endorsed by the publisher.
